# Evaluation of Different Restoration Combinations Used in the Reattachment of Fractured Teeth: A Finite Element Analysis

**DOI:** 10.1155/2018/8916928

**Published:** 2018-03-15

**Authors:** Nagihan Guven, Ozgur Topuz, İhsan Yikilgan

**Affiliations:** ^1^Department of Endodontics, Faculty of Dentistry, Baskent University, Ankara, Turkey; ^2^Department of Endodontics, Gazi University, Ankara, Turkey; ^3^Department of Restorative Dentistry, Gazi University, Ankara, Turkey

## Abstract

**Objective:**

The purpose of this study was to test different restoration combinations used for constructing fractured endodontically treated incisors by reattaching their fractured fragments.

**Methods:**

Seven types of 3-D FEM mathematical root canal-filled models were generated, simulating cases of (OB) reattaching fractured fragments; (CrPL) reattaching fractured fragments + ceramic palatinal laminate; (CmPL) reattaching fractured fragments + composite palatinal laminate; (CM) reattaching fractured fragments + coronal 1/3 of the root was filled using core material; (BP) reattaching fractured fragments + glass fiber post; (CP) composite resin restoration + glass fiber post; and (OC) composite resin restoration. A 100-N static oblique force was applied to the simulated teeth with 135° on the node at 2 mm above the cingulum to analyze the stress distribution at the tooth.

**Results:**

For enamel tissue, the highest stress values were observed in model BP, and the lowest stress values were observed in model CmPL. For dentine tissue, the highest stress concentrations were observed around the fracture line for all models.

**Conclusions:**

Reattachment of fractured fragments by bonding may be preferred as a restoration option for endodontically treated incisors; also, palatinal laminate decreases the stress values at tooth tissues, especially at the enamel and the fracture line.

## 1. Introduction

Restoration of endodontically treated teeth is an important issue in dentistry clinical practice. Endodontically treated teeth (ETT) have lower fracture resistance than vital teeth. This situation is caused by loss of substance (because of preexisting decay and endodontic access cavity preparation) and dehydration of teeth [1–3]. Many studies have corroborated that the ETT are less disposed to biomechanical failure when less dental hard tissue is removed for endodontic treatment [4]. The prognosis of ETT is also influenced by different parameters. These include the amount of hard tissue loss [5], presence of a minimum of 1.5–2.0 mm ferrule height preparation [6], and post and core material use [7].

The anterior teeth, especially the maxillary central incisors, are more commonly subjected to injury than the other teeth because of their position in the dental arch. Crown fractures represent the majority of dental trauma in permanent dentition (26–76% of dental injuries) [8–10]. Fractured anterior teeth are generally restored using direct composite resin or prosthetic restorations. Prosthetic restorations (especially metal based) may not provide adequate aesthetic harmony with the adjacent teeth. Additional disadvantages are that they require a significant tooth reduction during preparation and there can be inadequate periodontal adaptation.

Because of the excellent retention obtained with advanced bonding systems, the reattachment of tooth fragments has become a frequently used option for fractured teeth [8, 9]. The reattachment technique presents some advantages over composite and prosthetic restorations. This technique is generally faster and less complicated; more aesthetic restoration could be attained by conserving the original translucency and original shape, color, brightness, and contours as well as because the restored tooth is more resistant to staining and abrasion compared to resin restorations [11, 12]. Also, the incisal edge will be prevented. Furthermore, this restoration option can have the advantage of being a simple application technique, allowing the clinician to complete the procedure in a single visit.

Many studies have suggested techniques for reattaching the fractured tooth fragment to the remaining part. These include using a circumferential bevel before reattaching [13, 14], placing a chamfer at the fracture line after bonding [15], using a V-shaped enamel notch [16] or a groove with shoulder [17], and placing an internal groove or a superficial contour over the fracture line, while some authors have reported on the use of bonding with no additional preparation [18, 19]. Manju et al. [20] described a case of complicated fracture of the maxillary left immature permanent central incisor that was treated endodontically followed by esthetic reattachment of the fractured fragment using a glass fiber post. In addition, some authors have reported that porcelain laminate can be used to reinforce the fractured fragments that are bonded to each other. Andreasen et al. [21], in their experimental study, achieved the greatest fracture strength when a laminate veneer alone was used to restore the fractured incisal edge.

Considering the results of many published studies that examined the efficacy of restoration techniques for endodontically treated and fractured incisors, it has been revealed that the restoration technique significantly affects such restored teeth. However, to date, there is still no agreement in the literature about which material or technique can optimally restore endodontically treated teeth [22]. Moreover, there are no data about the effect of using the palatinal laminate restoration technique on the biomechanical behavior of restored teeth.

The aim of this study was to evaluate different restoration combinations, especially palatinal laminate, which is a novel approach used for constructing fractured endodontically treated incisors by reattaching their fractured fragments with finite element methods.

## 2. Materials and Methods

### 2.1. Preparation of Solid Model and Finite Element Models

**Figure 1:**
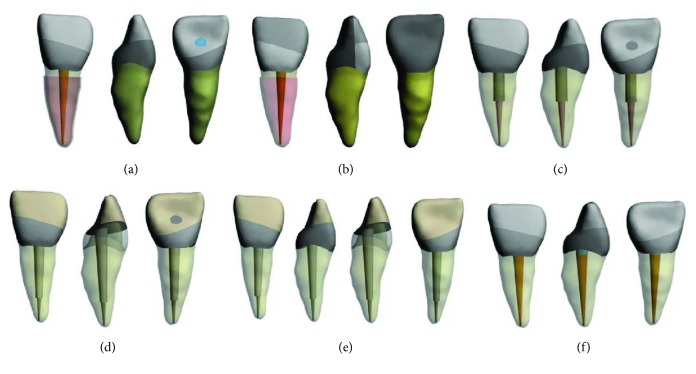
(a) Model OB, only the fracture fragment was bonded. (b) Model CrPL, the fractured fragment was bonded and a ceramic palatinal laminate was designated, and model CmPL, the fractured fragment was bonded and a composite palatinal laminate was designated. (c) Model CM, the fractured fragment was bonded and the coronal 1/3 of the root was filled using core material. (d) Model BP (bonding and post), the fractured fragment was bonded and a glass fiber post inserted into the root was designated. (e) Model CP, composite resin restoration and glass fiber post inserted into the root were designated without using the fractured fragment. (f) Model OC, composite resin restoration was designated without using the fractured fragment or adding any other applications.

This study was conducted using a three-dimensional (3-D) FE method, and a 3-D FEA mathematical model simulating an upper central incisor was created. After image acquisition of microcomputed tomography volume data, the geometric FEM model was performed in Mimics 10.01 (Materialise, Leuven, Belgium) by image thresholding. Then, the FEM model was obtained by importing the solid model into ANSYS 14.5 (ANSYS Inc. Southpoint, 275 Technology Drive, Canonsburg, PA, 15317, USA). Endodontic access cavities were prepared on the tooth models, and root canal fillings were positioned (Figures 1(a)–1(e)). ProTaper F3 gutta percha (Dentsply Maillefer, Switzerland), which is designed for use with the F3 file of the ProTaper rotary instrument for root canal filling, was adapted to the root canal system by modeling to 0.5 mm above the apex. Lamina dura (0.25 mm), periodontal ligament (0.25 mm), and cortical and cancellous bone (≥1.5 mm) were designed around the tooth root and matched with the tooth using ANSYS 14.5 (ANSYS Inc. Southpoint, 275 Technology Drive, Canonsburg, PA 15317, USA). For all models, cement was ignored. An oblique fractured line, which was identified on the software as a surface, was asymmetrically prepared on the coronal part of the model.

### 2.2. Restoration Options and Clinical Scenario

After combining the images of the procedures using Boolean expressions, based on seven different restoration options, seven different models of the endodontically treated maxillary incisors were developed. In the first clinical scenario, the fractured fragment was used for construction; in the second one, it was not used. Three-dimensional (3-D) FEM mathematical models simulated the following: (OB) only the fractured fragment was bonded; (CrPL) the fractured fragment was bonded, and a ceramic palatinal laminate was designated; (CmPL) the fractured fragment was bonded, and a composite palatinal laminate was designated; (CM) the fractured fragment was bonded, and the coronal 1/3 of the root was filled using core material; (BP) the fractured fragment was bonded, and a glass fiber post inserted into the root was designated; (CP) composite resin restoration and a glass fiber post inserted into the root were designated without using the fractured fragment; (OC) composite resin restoration was designated without using the fractured fragment or adding any other applications (Figure 1).

The palatinal lamina was modeled at a thickness of 2 mm, with limited interproximal contact, incisal edge, and palatinal gingival margin covering only the palatal region of the tooth in models CrPL and CmPL. Palatinal lamina post space was conically prepared and matched with another part, which was exactly the same on models BP and CP. The post cement layer, laminate cement layer, and bonding material on the fractured layer were identified as the surface instead of the thickness. Seven models designed with 7 different restorative approaches were prepared with Mimics Materialise software (MSC. Software, USA), which was transferred to ANSYS 14,5 (ANSYS Inc. Southpoint, 275 Technology Drive, Canonsburg, PA 15317, USA) in the STL format and meshed for analysis.

### 2.3. The Properties of Materials and Anatomical Structures

Young's modulus and Poisson's ratio, which describe the physical characteristics of each structure, were loaded into the software to identify the materials from which existing structures on the models prepared with Algor Fempro software were made (Table 1) [23–28]. Solid features were accepted as linearly resilient, homogenous, and isotropic in the program. It was assumed that the materials and anatomical structures were isotropic, linearly elastic, and homogeneous, except for the glass fiber post and dentine. The glass fiber post was considered as orthotropic so that it showed different mechanical properties along the fiber direction (*x* direction) and along the other two directions *y* and *z* directions (Table 2) [29]. The dentine of each model was assumed as orthotropic (Table 3) [30, 31]. In ANSYS, the postprocessing function was used to create a stress distribution diagram. We then analyzed the stress distributions and stress concentrations of the post and remaining dentin of the root for each modeled diameter of the post. For stress analysis, the required values of mechanical properties of the materials and anatomical structures included Young's modulus, Poisson's ratio, and density structures as shown in Tables 1–4. These values must be imported into the software to identify the physical differences of each part of the models.

### 2.4. Determination of Contact Surfaces on the Models

All interfaces between the modeled materials and anatomical structures were considered completely/tightly adhered.

### 2.5. Border Conditions

Zero motion and rotation were identified at six degrees of freedom from the side and upper surfaces of dental tissues.

### 2.6. Loading and Stress Analyses

**Figure 2:**
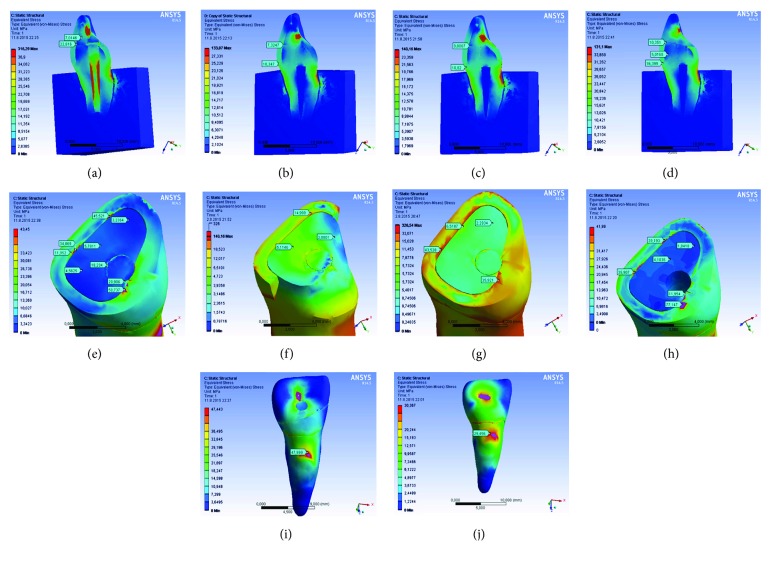
(a) At the tooth structure, the highest maximum von Mises stress values were observed in model BP for enamel. (b) The lowest stress values were observed in model. (c) For dentine tissue, the highest stress values were observed in model CrPL. (d) The lowest stress values were found in model CP. (e) On connection interfaces, at enamel, model CP had the highest stress values. (f) Model CrPL had the lowest stress values. (g) At dentine, the highest stress values were observed in model OB. (h) The lowest stress values were observed in model CM. (i) In root dentine, the highest stress values were observed in model BP. (j) The lowest stress values were observed in model CrPL.

To simulate the original occlusion, the models were constrained by a force of 100 N that was applied to an area over the cingulum on the palatal surface of the crowns of the models at a 135° angle to the long axis of the tooth (Figure 2). To calculate the stress distribution, the von Mises (equivalent stresses) energetic criterion was preferred [32]. The qualitative stress distribution analyses were recorded in this study using the von Mises criteria.

A view of a midsagittal and oblique-horizontal section from each model was provided to evaluate the stress distribution. Calculated numeric data obtained from each model were transformed into color images. For all structures, the highest von Mises stress values were recorded (Table 4).

## 3. Results

von Mises stresses at the 3 regions of interest were measured. The von Mises stress distribution in all models and extreme stress values are presented in Figure 2.  Table 5 shows the maximum von Mises stress values. At the tooth structure, the highest maximum von Mises stress values were observed in model BP for enamel, which is fractured fragment bonded and glass fiber post inserted into the designated root. However, the lowest stress values were observed in model CmPL for which fractured fragment was bonded and composite palatinal laminate was designated. For dentine tissue, the stress distribution pattern was similar to the models, except for model CrPL in which the highest stress values were observed. The lowest stress values were found in model CP as in model OB. There were stress concentration differences among the models at the tooth tissue on connection interfaces. On connection interfaces, at enamel, model CP had the highest stress values. It was anticipated that model CrPL (fractured fragment was bonded, and ceramic palatinal laminate was designated) had the lowest stress values. At dentine, the stress distribution patterns of all models were similar. The highest stress values were observed in model OB, and the lowest stress values were observed in model CM. In root dentine, the highest stress values were observed in model BP (fractured fragment was bonded, and glass fiber post inserted into the root was designated). The lowest stress values were observed in model CrPL (fractured fragment was bonded, and ceramic palatinal laminate was designated).

## 4. Discussion

In the present study, the FEA method was used to evaluate the pattern of stress distribution in different areas of endodontically treated central incisor models restored with different restorative approaches. The hypothesis of the study, which stated that the restoration approach would not affect the stress distribution of fractured endodontically treated central incisor teeth, was partially rejected.

The lowest values of the von Mises stress equivalents are observed at the connection interface of the models designed as fractured fragment bonded and ceramic or composite palatinal laminate designated. This result indicates that palatal laminate can protect the connection interface from stresses caused by occlusal forces. Similarly, in an experimental model using sheep incisors, Andreasen et al. [21] found that the fracture strength was equal to that of intact incisors. This is in contrast to fracture strengths of reattached enamel-dentin tooth fragments without porcelain laminates, which were only 50% of intact incisors. It is suggested that porcelain laminate veneers may be used to supplement fragment bonding, enhancing dental esthetics and function. The results of the study support our hypothesis for the beneficial usage of palatinal laminate, which is a novel approach to reinforce the fracture fragments. However, there is no study on the usage of palatinal laminate for any possible clinical scenario.

Anindya Bhalla et al. [33], in their recent case report, declared that when the fractured fragments are reattached with post for retention, post provides excellent retention with long-term stability of restored portion as in many previously reported cases [34, 35]. However, in this study, model BP, in which the fractured fragment was bonded and a glass fiber post was inserted into the root, is not superior to the other restoration alternatives. Even in the dental tissues and root surface, the highest stress values were observed in this model. The largest values of the von Mises stress equivalents indicate locations with the highest risk of fracture.

The use of core material (in model CM) has not shown a positive effect on the reduction of stress in the dental tissues, root surface, or connection interface. Moreover, at tooth tissues and the root surface in model CM, the von Mises stress value is higher than OB. This result may be from the elastic modulus of the core material being lower than that of the composite resin. Yikilgan and Bala [36] reported that materials with a low elastic modulus cause high stress levels, whereas materials with elastic moduli similar to those of dental tissues cause low stress levels.

At the root dentine, the highest stress values were observed in model BP (the fractured fragment was bonded, and a glass fiber post inserted into the root was designated). Although reinforcing the restoration with a fiber decreases the stress transmission and the fiber post structure accumulated more stress on its own body [37], the present study showed that the use of post material increased the stress values at the root dentine structure. Roscoe et al. [38] evaluated the effect of alveolar bone loss, post type, and ferrule presence on the biomechanical behavior of endodontically treated maxillary canines and found that 5.0 mm of bone loss significantly increased the stress concentration and strain on the root dentin. According to the evaluation of the maximum stress values, the maximum values occurred at the root dentine. Palatinal laminate is thought to be a safe approach for the tooth, which has alveolar bone loss. However, when 2i (direct composite resin restoration and glass fiber post) and OC (direct composite resin restoration) were compared at the connection interface and root surface in model 2i, the von Mises stress value was lower than CP. It was observed that the use of a fiber post reduces the stress buildup when fractured parts cannot be used.

These results demonstrated that palatinal laminate is an acceptable restorative approach for decreasing the stress concentration at the connection interfaces when autologous reattachment is preferred as a restorative option. When compared to laminate groups at enamel, the maximum von Mises stress value in model CrPL (in which the fractured fragment was bonded and a ceramic palatinal laminate was designated) is higher than that in model CmPL (in which the fractured fragment was bonded and a composite palatinal laminate was designated). The high elastic modulus of ceramic materials most likely causes this difference. Composite materials have mechanical properties that are similar to those of dentin. Consequently, they behave like monobloc units and withstand chewing forces [39].

The biomechanical behavior of endodontically treated teeth can be investigated with fracture strength tests and stress analysis methods. Fracture strength tests illustrate the maximum strength that the tooth endures until it becomes fractured. However, stress analysis methods can show long-term deformations that can occur in the dental tissues and restorative materials by masticator forces. In the stress analysis test, finite element analysis is considered ideal in terms of enabling reproducible situations and repeatable results. Because of these advantages, we used the finite element analysis in our study.

One of the limitations of FEA studies is that when the models are created, dentine has been modelled as a homogeneous/isotropic material, the structure of which is generally assumed to be homogeneous and isotropic [27, 40, 41]. In this study, the layer represented as dentine structure was considered orthotropic material. Therefore, the effects of dentinal tubules, intrapulpal hydrostatic pressure, and the elastic modulus gradient on the mechanical properties of dentine were not ignored [42].

Endodontically treated teeth are susceptible to biomechanical failure. The main factors of the tendency of such teeth to fracture are the loss of tooth tissue, altered physical properties of dentine, and altered proprioception/nociception, which cumulatively interact to influence the tooth loading and distribution of stresses [43, 44]. Therefore, the choice of the esthetic restorative treatment of fractured anterior teeth poses dilemmas to clinicians [45]. Direct composite resin restoration, fragment reattachment, and ceramic restorations (full crowns, laminate veneers, or ceramic fragments) are treatment options that may be preferred according to clinical situations or based on clinical decision. Two important criteria, such as the aesthetics and function of the diseased tooth, should be provided to perform an ideal treatment for a complicated crown fracture, which is dictated by various factors. With the significant development of adhesive systems and resin composites, the reattachment of tooth fragments is no longer a provisional restoration; instead, it is a restorative treatment with a favorable prognosis.

## 5. Conclusions

Within the limitations of this FEA study, the present study concluded the following:

- The model restored with only composite resin material had high stress values at tooth tissues. Hence, fragment reattachment is superior to composite restorations, although it may not match the intact tooth.
- The viability of tooth fragment reattachment along with post insertion as a restorative technique is unclear.
- Tooth fragment reattachment along with palatinal laminate is a viable technique.
- Palatinal laminate can safely be used as a novel restorative approach for teeth with alveolar bone loss.

In any possible clinical scenario, reattachment of the fractured tooth segment should be attempted as a priority. Reattachment of fractured fragments can be considered a good alternative treatment option when the fractured fragment is available. However, additional applications are required to prevent biomechanical failure of the tooth.

## Figures and Tables

**Table 1:** Mechanical properties of homogeneous and isotropic default layers.

Material	Elasticity modulus (GPa)	Poisson's ratio (*v*)	Reference
Trabecular bone	1.37	0.30	23
Cortical bone	13.7	0.30	23
Enamel	84.1	0.33	24
Periodontal ligament	6.89 × 10^−5^	0.45	25
Gutta percha	0.14	0.45	26
Composite	16	0.3	27
Ceramic	96	0.22	24
Resin core material	7	0.3	28

**Table 2:** Mechanical properties of orthotropic glass fiber post [29].

*E*_*x*_ (*GPa*)∗	37
*E*_*y*_ (*GPa*)∗	9.5
*E*_*z*_ (*GPa*)∗	9.5
*G*_*xy*_∗∗	0.27
*G*_*xz*_∗∗	0.34
*G*_*yz*_∗∗	0.27
*NU*_*xy*_∗∗∗	3.10
*NU*_*xz*_∗∗∗	3.50
*NU*_*yz*_∗∗∗	3.10

^∗^ *E_x_*, *E_y_*, and *E_z_* show the values of three-dimensional elasticity modules. ^∗∗^*G_xy_*, *G_xz_*, and *G_yz_* show orthogonal cutting module values in the plane. ^∗∗∗^*NU_xy_*, *NU_xz_*, and *NU_yz_* show the Poisson's ratios in the orthogonal plane.

**Table 3:** The orthotropic properties of dentin [30, 31].

*E*_11_ (*GPa*)^∗^	25
*E*_33_ (*GPa*)^∗^	23.2
*v*_21_^∗∗^	0.45
*v*_31_^∗∗^	0.29
*G*_12_(*GPa*)^∗∗∗^	8.6
*G*_23_(*GPa*)^∗∗∗^	9.4

^∗^ *E*: Young's modulus; ^∗∗^*v*: Poisson's ratio; ^∗∗∗^*G*: shear modulus.

**Table 4:** Density values of layers.

Material	Density (10^−6^ kg/mm3)
Trabecular bone	1.3
Cortical bone	1.3
Enamel	2.6
Dentine	2.1
Periodontal ligament	1.04
Gutta percha	0.9
Composite	2.1
Ceramic	2.4
Resin core material	2.24
Glass fiber post	2.5

**Table 5:** Maximum von Mises stress values for tooth structures, connection interfaces, and root dentin (MPa).

Clinical scenario	Maximum von Mises stress values for tooth structures (MPa)		Maximum von Mises stress values for (MPa)		Maximum von Mises stress values for root dentin (MPa)
*(1) Groups that used fractured fragment for construction*					
OB	Enamel	17.555	Enamel	43.528	30.432
	Dentine	5.5604	Dentine	6.5187	
CrPL	Enamel	10.82	Enamel	14.999	29.456
	Dentine	9,0007	Dentine	5,1146	
CmPL	Enamel	10.347	Enamel	18.442	31.256
	Dentine	7.3247	Dentine	6.0642	
CM	Enamel	20.929	Enamel	39.193	40.146
	Dentine	6.4163	Dentine	4.1036	
BP	Enamel	22.816	Enamel	38.619	47.999
	Dentine	7.0146	Dentine	5.5755	
*(2) Groups that did not use fractured fragment for construction*					
CP	Enamel	17.263	Enamel	33.651	40.127
	Dentine	6.5185	Dentine	5.096	
OC	Enamel	16.399	Enamel	45.521	42.04
	Dentine	5.168	Dentine	5.7811	
